# Vitiligo Adverse Event Observed in a Patient With Durable Complete Response After Nivolumab for Metastatic Renal Cell Carcinoma

**DOI:** 10.3389/fonc.2019.01033

**Published:** 2019-10-09

**Authors:** Emilien Billon, Jochen Walz, Serge Brunelle, Jeanne Thomassin, Naji Salem, Mathilde Guerin, Cecile Vicier, Slimane Dermeche, Laurence Albiges, Florence Tantot, Soazig Nenan, Geraldine Pignot, Gwenaëlle Gravis

**Affiliations:** ^1^Department of Medical Oncology, Institut Paoli-Calmettes, Marseille, France; ^2^Department of Urology, Institut Paoli-Calmettes, Marseille, France; ^3^Department of Radiology, Institut Paoli-Calmettes, Marseille, France; ^4^Department of Biopathology, Institut Paoli-Calmettes, Marseille, France; ^5^Department of Radiotherapy, Institut Paoli-Calmettes, Marseille, France; ^6^Department of Cancer Medicine, Gustave Roussy, Villejuif, France; ^7^Research Department, UNICANCER, Paris, France; ^8^Centre de Recherche en Cancérologie de Marseille, INSERM UMR1068; CNRS UMR7258, Institut Paoli-Calmettes, Aix Marseille Université, Marseille, France

**Keywords:** renal cell carcinoma, nivolumab, immunotherapy, complete response, immune adverse events, vitiligo, thyroid dysfunction, nephrectomy

## Abstract

**Background:** Renal cell carcinoma is the third most prevalent urological cancer worldwide and about 30% of patients present with metastatic disease at the time of diagnosis. Systemic treatments for metastatic renal cell carcinoma have improved recently. Vascular endothelial growth factor targeting therapies were the previous standard of care. However, immune checkpoint inhibitors used in second line therapy have now been shown to improve patient survival. We report a case of metastatic renal cell carcinoma with nivolumab as a second-line therapy after progression with tyrosine kinase inhibitor therapy. Unusual adverse events in metastatic renal cell carcinoma, such as vitiligo, were observed in this patient who developed a remarkable documented pathological complete response to his renal tumor.

**Case presentation:** A 60-year-old caucasian male was diagnosed with a pulmonary metastatic clear cell renal cell carcinoma. Sunitinib was used as first line treatment without success. He received nivolumab in second-line treatment. He developed several immune-related adverse events, most notably vitiligo. The patient had a radiological complete response on metastatic sites, with a significant decrease of renal tumor volume and underwent cytoreductive nephrectomy after 2 years of treatment, confirming the pathological complete response. The patient remains disease-free for 10 months without further systemic therapy after nivolumab discontinuation.

**Conclusions:** Pathological complete response with nivolumab in metastatic renal cell carcinoma is rare. This case further highlights the potentially predictive role of immune-related adverse events during nivolumab therapy for metastatic renal cell carcinoma and raises questions concerning the role of nephrectomy after immune checkpoint inhibitor therapy. Further studies are needed to better identify predictive factors for treatment response to immunotherapy in metastatic renal cell carcinoma, and to better understand the role of nephrectomy after nivolumab treatment.

## Background

Renal cell carcinoma is the third most prevalent urological cancer worldwide with 380,000 new cases diagnosed every year (1). Of these, about 30% of patients present with metastatic disease at the time of diagnosis (2). Over the past decade, remarkable progress has been made in the treatment of metastatic clear cell renal cell carcinoma. Tyrosine kinase inhibitors (TKIs) and immune checkpoint inhibitors have been shown to improve survival (3–5), though immune checkpoint inhibitors were developed as a second-line treatment after TKI failures (6). Furthermore, the administration of immune checkpoint inhibitors therapy in untreated metastatic clear cell renal cell carcinoma demonstrated improved survival for patients with intermediate and poor-risk diseases [CheckMate-214 trial (7)], while the combination of checkpoint inhibitors plus vascular endothelial growth factor receptor inhibition improved both overall survival (OS) and progression free survival (PFS) over TKI therapy alone (8, 9).

Based on the phase III Checkmate 025 study, the PD-1 checkpoint inhibitor nivolumab was approved by the U.S. Food and Drug Administration and the European Medicines Agency for advanced metastatic clear cell renal cell carcinoma patients previously treated with TKIs. Nivolumab demonstrated benefits to both OS and the objective response rate (ORR) when compared to everolimus (6), while the side-effects (grade 3–4 Adverses Events 19 vs. 37%, respectively) and quality of life scores also favored patients treated with nivolumab. Nivolumab treatment improved median OS by 5.4 months, with an ORR of 25% and a complete response rate of 1% (6). Nivolumab's safety profile is different from conventional therapy and was responsible for several immune-related adverse events (irAEs), such as interstitial pneumonia, diarrhea, autoimmune hepatitis, and endocrine dysfunction (6, 10).

We report a case of metastatic renal cell carcinoma in a clinical trial (GETUG–AFU 26-NIVOREN, NCT03013335) with nivolumab as a second-line therapy after progression with TKI therapy. Unusual AEs in renal cell carcinoma were observed, and the patient developed a remarkable documented pathological complete response to his primary renal cell carcinoma.

## Case Presentation

In February 2015, a 60-year-old Caucasian male with a seven-month history of chronic cough and macroscopic hematuria and no history of tobacco use was diagnosed with a pulmonary metastatic clear cell renal cell carcinoma. The patient also had a personal history of hyperthyroidism (Graves' disease, laboratory assays were performed before the start of any antitumoral therapy and indicated normal thyroid function), which was originally treated in 2013 with neomercazole, which was then replaced by 100 μg per day of levothyroxine. A computerized tomography (CT) scan revealed a 110 mm mass on the left kidney, as well as the presence of bilateral pulmonary lesions. Analysis of the kidney tumor biopsy further revealed a clear cell renal carcinoma, Fuhrman grade II.

**Figure 1:**
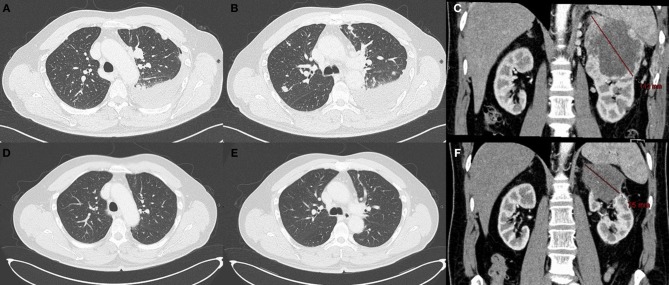
CT scan after sunitinib therapy and while under nivolumab Pulmonary metastasis **(A,B)** and renal lesion **(C)** after progression under sunitinib. Radiological complete response of the pulmonary metastasis **(D,E)** under nivolumab therapy at 6 months. The CT scan showed only a 75 mm mass on the left kidney **(F)**.

In March 2015, the patient was randomized in the CARMENA trial (NCT00930033) and received sunitinib (50 mg per day), without nephrectomy. By February 2016, the patient's disease had progressed with new lung, pleural (Figures 1A–C), and bone metastases, and he was therefore offered inclusion in the GETUG–AFU 26-NIVOREN trial (NCT03013335). After inclusion, the patient received anti-PD-1 therapy with nivolumab (3 mg/kg every 2 weeks) in March 2016. Upon the third injection of nivolumab, the patient developed lower back pain and required the use of morphine whose perfusion duration was then increased for each subsequent administration.

After 3 months of treatment, the patient developed clinical and biological signs of hyperthyroidism with palpitations and tremors associated with low TSH (0.005 mUI/L) serum levels and high fT3 and fT4 (11 pmol/L and 39 pmol/L, respectively) serum levels. Thyroid scintigraphy did not detect any ^123^I fixation, and the levels of anti-thyroid peroxidase and anti-thyroglobulin antibodies had not increased, thereby confirming the presence of a nivolumab-related, thyroid-related adverse event. Propranolol 120 mg per day was prescribed to counter symptoms caused by the hyperthyroidism. Because it was not being efficient enough, Neomercazole, 60 mg per day, was introduced and was quickly stopped for clinical and biological normalization; this was followed by the reintroduction of hormone replacement therapy.

**Figure 2:**
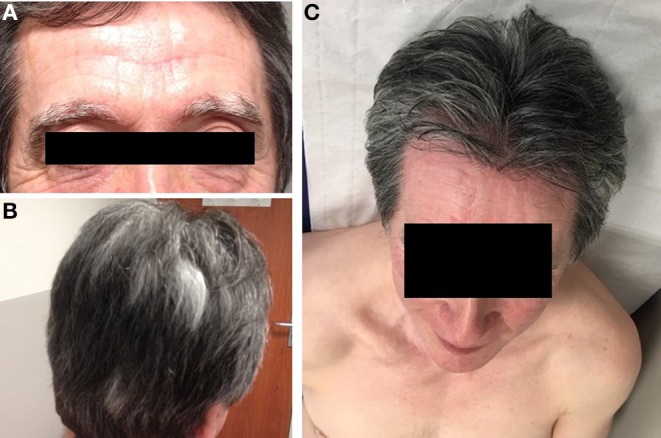
Vitiligo lesions. Depigmentation of eyebrows **(A)**, hair **(B)**, and skin **(C)** observed after 8 months of treatment with nivolumab. Depigmentation affected the whole skin but preferentially the chest.

We observed a partial response after 3 months of treatment and complete response in the lungs (Figures 1D–F) and bone after 6 months. After 8 months of treatment with nivolumab, the patient developed a depigmentation of his eyebrows and hair that was suspected to be indicative of vitiligo (Figures 2A,B). As nivolumab was maintained the vitiligo spread further, affecting the eyelashes and skin over the entire body (Figure 2C).

**Figure 3:**
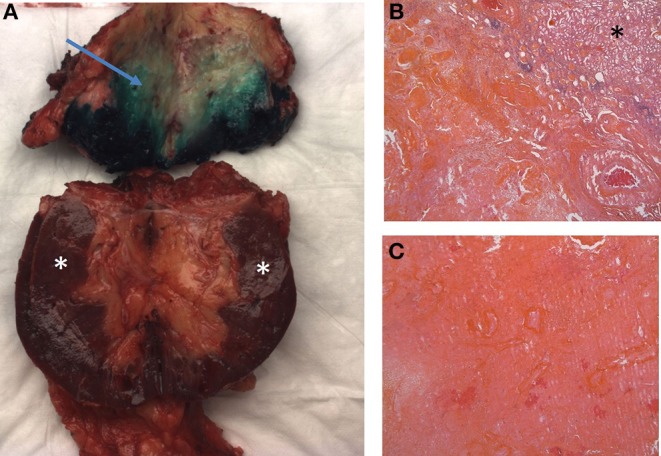
Macroscopic and microscopic examination. Macroscopic examination **(A)**: fibrosis alterations on upper pole of the kidney (arrow) Microscopic examination **(B,C)**: fibrosis alterations with calcifications and without residual tumor cells. Stars indicate normal parenchyma.

After 2 years of treatment, complete response was confirmed in the lungs by CT scan, with only the primary lesion of the left kidney remaining (65 vs. 110 mm at the diagnosis). The possibility of a cytoreductive nephrectomy was discussed with the patient and with a multidisciplinary urologic oncology team. In May 2018, a partial nephrectomy was initially planned but surgeons described difficulties in finding dissection planes because of major adhesions and inflammatory reactions in the kidney and surrounding tissue. The extent of surgery has been changed during the procedure because it was impossible to identify tumor boundaries (switch from partial to radical nephrectomy, in order to avoid potential positive surgical margins). Pathological analysis (Figure 3) revealed a lesion of 6 cm at the superior pole of the kidney with fibrosis, focally calcified, and without residual tumor cells.

The final nivolumab administration was performed on May 2018. As of the time of last follow up (April 2019) the patient was in complete response.

## Discussion

We report a case of metastatic clear cell renal cell carcinoma with histological complete response after nivolumab administration as a second-line therapy, which resulted in an uncommon (in renal cell cancer) vitiligo side effect.

Immune-related adverse events commonly result from the use of immune checkpoint inhibitors. Vitiligo itself is an acquired pigment disorder in which depigmented macules result from the loss of melanocytes from the involved regions of skin and hair. Vitiligo occurs worldwide with an estimated prevalence rate of 0.5–1% and could be associated with several autoimmune diseases such as thyroid disease, rheumatoid arthritis, and type 1 diabetes (11). However, the incidence of vitiligo in the immune checkpoint inhibitor-treated population is not precisely known. In a meta-analysis of immunotherapy in patients with stage III and IV melanomas, the cumulative incidence of vitiligo was 3.6% (95% CI [2.64, 4.78]) and was significantly associated with a decreased risk of disease progression (one-half) and death (one-quarter), for patients with vitiligo compared to patients without vitiligo (12). A systematic review and meta-analysis of randomized clinical trials investigated the toxicity profile of approved anti-PD-1 monoclonal antibodies in solid tumors (nine randomized trials and 5,353 patients were included). Cases of vitiligo were reported in five out of the nine studies and only among patients with a diagnosis of metastatic melanoma with a strong correlation between irAEs and improvement in ORR and survival. A pooled analysis of 576 patients treated with nivolumab found that irAEs of any grade were associated with higher ORRs without any difference in PFS (13).

Thyroid dysfunction, such as the thyroid-related adverse events observed with our patient, was reported to be an independent predictive factor of favorable outcomes for OS and PFS in a prospective trial with 58 patients with non-small cell lung cancer treated with PD-1 blockade (14). In our case, the patient had a history of Grave's disease and developed thyroid dysfunction. However, laboratory assays and the scintigraphy were not compatible with Graves' disease but confirmed nivolumab induced adverse event. Experience concerning the impact of checkpoint inhibitors on patients with preexisting autoimmune diseases is limited because these patients were usually excluded from clinical trials. Two retrospective studies described the use of PD-L1 inhibitors for metastatic melanoma in patients with pre-existing autoimmune disorders (15, 16). In these two series, of, respectively, 52 and 17 patients, a flare of the pre-existing autoimmune disorder was observed in 40% of patients. Response rates were above 30% in the two population and were in the range expected from clinical studies in patients without preexisting immunity disorders (21–32% for pretreated patients (17, 18) and 33–43% for untreated patients (19, 20). In a cohort of 56 patients treated with immune checkpoint inhibitors for non-small cells lung cancer, 23% developed flare of their pre-existing immune disorder, with a response rate of 22%. In this study, the incidence of irAEs was like reported rates in clinical trials where patients with immune disorders were excluded (21).

Our patient developed lumbar pain during the third nivolumab infusion. Lower back pain has been previously described as a possible rheumatic irAE, and a recent publication observed a high ORR to anti-PD1 correlation in the melanoma subgroup in association with rheumatic irAEs (22).

Certain HLA genotypes, such as HLA DQ2/DQ8 or HLA DQA1, might be associated with immune disorders (23) and could be consequently associated with irAEs (24) or tumor response to ICIs. However, these genotypes were not detected in our patient after HLA typing. Recent studies suggest that HLA expression may affect the response to immune checkpoint inhibitors in advanced melanoma (25) and Hodgkin's lymphoma (26). Patients with MHC class II-positive and MHC class I-low expression tumors might have better responses and improved OS.

The role of nephrectomy is still unclear for patients who have a complete response to nivolumab in renal cell carcinoma. Approximately 90% of patients in the Checkmate 025 trial had a prior nephrectomy before systemic therapy, yet only a few (1%) had a complete response to treatment (6). Two other cases of total nephrectomy after radiological complete response with nivolumab were also described (27, 28), where both cases observed complete pathological responses without any viable malignant cells. These two cases, such as our, indicate that total nephrectomies could be safely carried out for metastatic clear cell renal cell carcinoma after nivolumab therapy, however, in our case, a partial nephrectomy was impossible due to significant post-immunotherapy fibrosis. Also, it is important to note that, in the case of pathological complete response, a biopsy is required before surgery in order to avoid an unnecessary nephrectomy.

The question of nivolumab discontinuation remains unanswered in metastatic clear cell renal cell carcinoma for patients treated for 2 years with pathological complete response. In metastatic melanomas, retrospective and prospective data indicated excellent results with immune checkpoint inhibitor therapy, even after discontinuation. In the phase III Checkmate 067 study, 159/314 patients treated by the combination nivolumab + ipilimumab were still alive at 4 years, and 113 (71%) of them are free from study treatment and have never received subsequent systemic therapy (29). For patients who received nivolumab alone, 138/316 patients were still alive after 4 years, and 69 stopped the treatment for any reason and never received other systemic therapy. In contrast, in the phase III Checkmate 017 and Checkmate 057 studies, 20/83 patients responding to nivolumab for non-small cell lung cancer maintained an objective response after 3 years (26/418 patients continued nivolumab at 3 years) (30). Furthermore, in a retrospective study of 19 patients with non-small cell lung cancer responding to immune checkpoint inhibitor therapy, for those who stopped immune checkpoint inhibitor treatment due to AEs (31) the median PFS after discontinuation depended on the confirmed response during administration, as PFS was not reached for partial response patients (4/19) vs. 4.9 months for stable patients (12/19). Additionally, in a retrospective analysis of 262 patients treated with immune checkpoint inhibitor therapy in phase I studies for all types of cancer, immunotherapy was discontinued in 39 cases for reasons other than progression, while 24 patients were still responding to treatment and 39 were in complete response (32).

Nivolumab discontinuation was not documented in mCCRCC, and the decision, in our case, was made in concert with the patient.

## Conclusion

We reported herein a case of metastatic clear cell renal cell carcinoma with radiological and pathological complete response after nivolumab therapy and the associated irAEs. This case further highlights the potentially predictive role of irAEs during nivolumab therapy for mCCRCC. Further studies are needed to better identify predictive factors for treatment response to immunotherapy in metastatic renal cell carcinoma, and to better understand the role of nephrectomy after nivolumab treatment.

## Ethics Statement

Written informed consent was obtained from the individual(s) for the publication of any potentially identifiable images or data included in this article.

## Author Contributions

GG and EB: conception and design and manuscript writing. EB, JW, SB, JT, NS, MG, CV, SD, LA, FT, SN, GP, and GG: final approval. JT: pathological explorations. SB: radiological exploration. GG, JW, and GP: patient's management.

### Conflict of Interest

GP serves as board member for BMS. GG and SD receive travel grants from BMS and Pfizer. SD receives travel grants from AMGEN and ASTRAZENECA. JW participate to speaker's bureau for ASTRAZENECA, JANSSEN, TAKEDA, and BLUE EARTH DIAGNOSTICS. The remaining authors declare that the research was conducted in the absence of any commercial or financial relationships that could be construed as a potential conflict of interest.

## Abbreviations

CT: Computerized Tomography
ICI: Immune Checkpoint Inhibitor
irAEs: immune-related Adverse Events
HLA: Human Leukocyte Antigen
mCCRCC: metastatic Clear Cell Renal Cell Carcinoma
ORR: Objective Response Rate
OS: Overall Survival
PD-1: Programmed Death 1
PDL-1: Programmed Death Ligand 1
PFS: Progression Free Survival
TKI: Tyrosine Kinase Inhibitor.
